# Patient-specific computational simulation for thoracic endovascular aortic repair in patients with Stanford type B aortic dissection

**DOI:** 10.3389/fcvm.2026.1821123

**Published:** 2026-08-19

**Authors:** Xinbo Liu, Haibo Yang, Lei Yang, Chao Li, Yaojie Wang, Bo Li, Youjin Li

**Affiliations:** 1 Department of Cardiovascular Surgery, People’s Hospital of Ningxia Hui Autonomous Region, Ningxia Medical University, NingXia, Yinchuan, China; 2 The Third Clinical College of Ningxia Medical University, NingXia, Yinchuan, China

**Keywords:** computational fluid dynamics, computational simulation, Stanford type B aortic dissection, thoracic endovascular aortic repair, three-dimensional reconstruction

## Abstract

**Background:**

The accuracy and clinical utility of patient-specific computational simulations for thoracic endovascular aortic repair (TEVAR) planning in Stanford type B aortic dissection (TBAD) patients remain insufficiently validated.

**Objectives:**

This study aimed to develop and validate the safety and efficacy of a patient-specific computational simulation framework tailored for TEVAR in patients with TBAD.

**Methods:**

This prospective, observational, multicenter study enrolled 153 consecutive patients with TBAD undergoing TEVAR. Patients were stratified into two groups: the simulation group (*n*=72) and non-simulation group (*n*=81). For the simulation cohort, personalized three-dimensional models of the stent frame and covered graft were constructed based on preprocedural computed tomography angiography, followed by computational simulation. The predictive performance of the simulation was evaluated by comparing simulated hemodynamic parameters with postprocedural measurements. The primary endpoint was the procedural success rate.

**Results:**

The overall cohort had an average age of 67.1 ± 9.1 years, with 66.0% being male. The simulation group showed a higher procedural success rate (100.0% vs. 95.1%, *P* = 0.042), lower rates of endoleak (0% vs. 9.9%, *P* = 0.007) and stent-related rupture (0% vs. 4.9%, *P* = 0.002), and a lower complete false lumen thrombosis rate (61.1% vs. 79.0%, *P* = 0.015) compared to the non-simulation group. Notably, postprocedural hemodynamic metrics (including maximum pressure gradient, peak velocity, and wall shear stress) were significantly improved in the simulation group (all *P* < 0.001).

**Conclusions:**

Patient-specific computational simulation of TEVAR was associated with postprocedural hemodynamic profiles that closely matched simulated predictions, and showed favorable periprocedural trends and potential utility in optimizing pre-TEVAR planning. These findings are preliminary and require validation in randomized controlled settings.

## Introduction

Aortic dissection (AD) is a life-threatening vascular catastrophe characterized by the tear in the aortic intima-media layer, triggered by factors such as aortic medial degeneration, trauma, or hypertension (1). Clinical manifestations typically include severe, progressive chest pain, with severe cases progressing to shock or fatal aortic rupture (1). For Stanford type B aortic dissection (TBAD), thoracic endovascular aortic repair (TEVAR) is the first-line therapeutic modality (2). Prior clinical evidence has demonstrated that TEVAR outperforms open surgical repair in terms of lower early mortality and major complication rates (3, 4).

However, TEVAR remains challenging in TBAD management: procedures involving the aortic arch and its critical branches are associated with higher mortality, and incomplete false lumen (FL) thrombosis post-procedure is common, which elevates the risk of adverse long-term outcomes (5, 6). This is largely attributed to the fact that most aortic stent-grafts only cover the proximal entry tear, leaving distal re-entry tears untreated—resulting in persistent communication between the true lumen (TL) and FL. Thus, accurate identification of patients at risk of such adverse events is clinically imperative. A promising strategy to address this gap is patient-specific computational simulation. With the advancement of computational fluid dynamics (CFD), preprocedural analysis of the aortic anatomy and TEVAR simulation using computed tomography angiography (CTA) data have become feasible (7). However, prospective validation of its clinical utility and predictive accuracy is lacking.

In this study, we aimed to validate the efficacy of patient-specific computational simulation by performing three-dimensional (3D) reconstruction of preprocedural CTA images, simulating TEVAR procedures, and comparing the simulated results with postprocedural CFD-derived hemodynamic metrics.

## Methods

### Study participants

This was a prospective, observational, multicenter study enrolling consecutive patients with TBAD who were scheduled for TEVAR. The enrollment period spanned from May 2024 to October 2025. All patients underwent pre- and postprocedural CTA in accordance with standardized clinical care protocols. To ensure cohort homogeneity regarding chronicity, all included patients were treated strictly during the acute phase of Stanford type B aortic dissection, with the time from symptom onset to TEVAR intervention being within 14 days. Inclusion criteria: 1) confirmed diagnosis of TBAD based on clinical manifestations and CTA findings; 2) presence of aortic rupture and/or visceral malperfusion; 3) planned TEVAR as the primary therapeutic intervention. Exclusion criteria: 1) severe hepatic or renal dysfunction [defined as Child-Pugh class C liver disease or estimated glomerular filtration rate (eGFR) < 30 mL/min/1.73 m^2^]^1^; 2) concurrent Stanford type A aortic dissection, retrograde aortic dissection, or hematoma involving the left common carotid artery and its proximal segment; 3) comorbid thoracic aortic intramural hematoma, thoracic aortic aneurysm, or penetrating aortic ulcer; 4) preprocedural CTA demonstrating dissection involvement of aortic arch branches (excluding the left subclavian artery); 5) diagnosis of Marfan syndrome or other heritable connective tissue disorders. After patient screening, a total of 153 patients were included in this study, including 72 cases in the simulation group and 81 cases in the non-simulation group. Patient allocation into the two treatment cohorts was performed via a sequential, time-dependent strategy to minimize selection bias. Specifically, consecutive patients enrolled during the initial study phase (from May 2024 to February 2025) were assigned to the non-simulation group, during which the multi-center 3D simulation pipeline was undergoing software standardization and infrastructure setup. Following complete workflow optimization and validation of the simulation framework, consecutive patients treated during the subsequent phase (from March 2025 to October 2025) were uniformly assigned to the simulation group. This allocation was entirely independent of operator preference, center-specific protocols, or baseline patient anatomical complexity. Additionally, this study was conducted in adherence to the Declaration of Helsinki. Ethical approval was obtained from the institutional review boards of all participating centers. Written informed consent was obtained from all eligible patients or their legal representatives prior to enrollment.The computational pipeline in the simulation group was initiated immediately after preprocedural CTA acquisition and completed in parallel with standard preoperative preparation; the median processing time was 4–6 h, and no patient experienced a clinically significant delay in TEVAR intervention due to awaiting simulation results. In the non-simulation group, no simulation-related waiting period was imposed, and the time from CTA to TEVAR was determined solely by standard clinical logistics. The median time from CTA acquisition to TEVAR was comparable between the two groups, with no statistically significant difference (*P* > 0.05), ensuring that differential waiting times did not introduce bias in outcome interpretation.

### Model reconstruction and mesh generation

**Figure 1:**
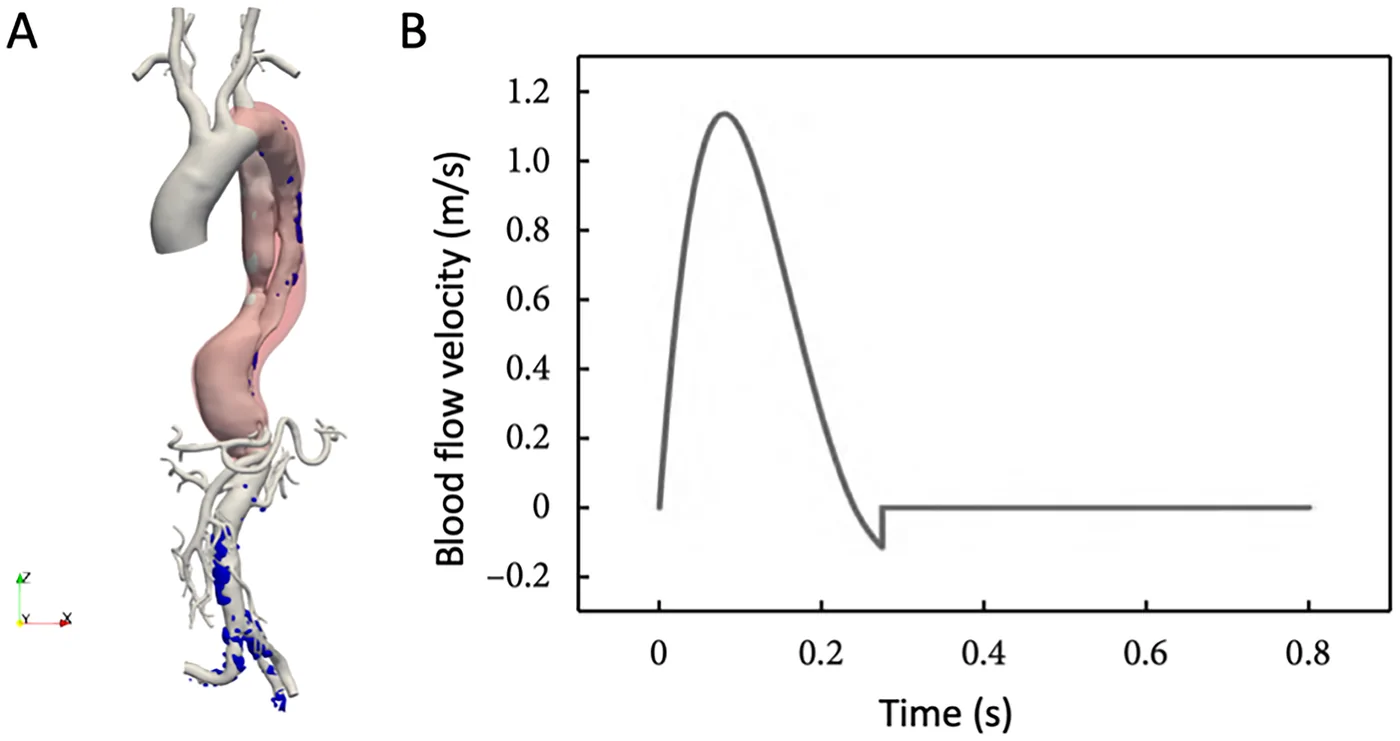
Model reconstruction and boundary condition setup. **(A)** Three-dimensional geometric reconstruction of the native. The blue region indicates the healthy aortic arch branches (brachiocephalic trunk, left common carotid artery, and left subclavian artery) with preserved patency prior to stent deployment. **(B)** Time point selected at the peak systolic phase during rapid ejection in the cardiac cycle.

(1) Patient-Specific Stent-Graft Design: a planar M-shaped 8-peak stent frame was designed using Rhino 7 (Robert McNeel & Associates, Seattle, WA, USA), with geometric parameters tailored to TL and FL anatomical features derived from the patient's preprocedural CTA. A 3D solid model of the stent frame was constructed via the tube tool in Rhino 7, where the stent length was matched to the actual main stent-graft length used intraprocedurally, and the stent diameter was oversized by 10%–15% relative to the target aortic diameter (to ensure radial apposition and seal). The covered graft membrane was then designed to conform to the stent frame's length and diameter. All final stent-graft models were exported in stereolithography (STL) format for subsequent integration.
(2) 3D Aortic Reconstruction (Figure 1A): Preprocedural CTA data (DICOM format) were imported into 3D Slicer (Version 5.4, Harvard Medical School, Boston, MA, USA) for semi-automated segmentation. Optimal Hounsfield unit thresholds and manual editing tools were applied to precisely delineate the TL and FL while excluding non-target structures. The segmented aortic model was exported as an STL file and further refined in Materialise Magics 24.0 (Materialise, Leuven, Belgium) to smooth surface irregularities, repair mesh defects, and remove artifacts introduced by CTA image noise. Crucially, all secondary distal re-entry tears and communications between the channels identifiable on CTA were fully preserved during segmentation to ensure hemodynamic accuracy.
(3) Mesh Generation and Format Conversion: The reconstructed AD model, patient-specific stent frame, and covered graft membrane were imported into ANSYS ICEM CFD 19.0 (ANSYS Inc., Canonsburg, PA, USA) for tetrahedral unstructured mesh generation. Meshes were exported in MSH format and converted to INP format using HyperMesh 2020 (Altair Engineering, Troy, MI, USA). To ensure mesh independence, a grid convergence index analysis was performed, yielding a final optimized mesh configuration consisting of approximately 4.5–5.5 million tetrahedral elements, depending on patient-specific geometry. To accurately capture the steep velocity gradients near the endografts, five prism inflation layers with a growth rate of 1.2 were generated along the vascular walls, maintaining a maximum cell skewness below 0.82 to guarantee geometric quality.

### Material property settings

The stent frame was modeled as nitinol using Abaqus CAE 2021 software (Dassault Systèmes, Vélizy-Villacoublay, France). To ensure a uniform modeling pipeline, the simulation treated all endografts under standardized nitinol properties, independently of the specific commercial brand used (Valiant, Gore TAG, or Ankura). Nitinol, capable of transitioning between austenite and martensite phases at specific temperatures, was selected as the preferred material for the stent frame due to its superelastic properties. In this study, the stent frame was defined as an isotropic, homogeneous, and incompressible material with a density of 6,450 kg/m^3^ and a Poisson's ratio of 0.3. This geometric conformation step was performed solely to predict the deployed configuration of the virtual stent-graft; it did not constitute a full fluid-structure interaction (FSI) analysis. No FSI analysis was performed in this study, and all hemodynamic calculations assumed rigid vascular walls.

### CFD analysis

The AD model was imported into Fluent software (ANSYS Inc., Canonsburg, PA, USA) for CFD analysis. All hemodynamic simulations were performed under a rigid-wall assumption, with the aortic wall modeled as rigid and the stent-graft geometry treated as a fixed boundary. The following physicochemical parameters were applied (8): blood was modeled as an incompressible Newtonian fluid with a density of 1,050 kg/m^3^ and a dynamic viscosity of 0.0035 Pa·s. Given that blood flow in the aorta is rarely turbulent, the flow was assumed to be laminar (8). The inlet at the ascending aorta was defined with a velocity boundary condition representing the pulsatile blood flow waveform over one cardiac cycle with a standardized scale matching the patient's mean heart rate. The outlets at the descending aorta and three branch vessels were assigned three-element Windkessel model pressure boundary conditions to mimic distal vascular resistance. The governing Navier-Stokes equations were solved using a second-order upwind scheme, and the computational convergence criteria for each time step (10^−3^ s) were strictly defined by a normalized residual threshold of less than 10^−5^ for both continuity and momentum equations. Fixed constraints were applied to the vessel walls at the ascending aortic inlet, descending aortic outlet, and branch vessels based on patient-specific aortic model parameters.

The peak systolic time point during the rapid ejection phase of the cardiac cycle (Figure 1B) was selected for analysis. After replacing the diseased segment of the aorta with a stent frame, the maximum pressure gradient (PG_max_), velocity (V_max_) and wall shear stress (WSS_max_) within FL at the level of the distal landing zone were recorded. Using the same methodology, postprocedural CTA images after TEVAR were reconstructed into a 3D model and processed via CFD to obtain the corresponding PG_max_, V_max_ and WSS_max_ at the same anatomical level. A comparison was made between the pre- and post-TEVAR results, and the discrepancy in hemodynamic parameters derived from CFD simulations was quantified to evaluate the validity of the computational TEVAR simulation. Crucially, to eliminate potential observer bias and ensure objectivity, the core-laboratory computational engineers responsible for performing the pre-TEVAR virtual simulations and postprocedural CFD evaluations were completely blinded to the clinical group allocations, commercial stent brands utilized, and in-hospital outcome endpoints.

### Procedural details

All TEVAR procedures were performed under local anesthesia or general anesthesia. The standardized procedural workflow was as follows: the left radial artery was punctured, followed by administration of intravenous heparin (70–100 U/kg body weight) to maintain an activated clotting time (ACT) of 250–300 s. A 6F vascular sheath was inserted into the radial artery, and a 5F pigtail catheter was advanced to the ascending aorta for multi-projection angiography (anteroposterior, lateral, and oblique views). This step was used to precisely assess: location and size of the primary entry tear; distance from the tear to the left subclavian artery ostium; involvement of aortic arch branches or distal aortic branches. Then, the right femoral artery was accessed percutaneously using the Seldinger technique, and a 10F vascular sheath was inserted. A super-slippery hydrophilic guidewire was used to navigate a pigtail catheter to the ascending aorta; the catheter was then exchanged for a stiff guidewire advanced to the aortic sinus to provide stable support for stent delivery. A self-expanding covered stent-graft was delivered over the stiff guidewire to the target position, with deployment controlled to fully cover the primary entry tear. Because all patients possessed a healthy proximal landing zone length of ≥15 mm relative to the left subclavian artery (LSA), the self-expanding covered stent-graft was consistently delivered and deployed with a standalone Zone 3 landing strategy, fully covering the primary entry tear while completely preserving LSA patency. No LSA coverage occurred, and no adjunctive procedures including carotid-subclavian bypass, transposition, branched endografts, or fenestration techniques were required or performed in either cohort. Clinical stent-graft models used included: Valiant^™^ (Medtronic, Minneapolis, MN, USA); Gore TAG® (Gore & Associates, Flagstaff, AZ, USA); Ankura® (Lifetech Scientific, Shenzhen, China). After confirming satisfactory angiographic results, the delivery system was withdrawn, and vascular sheaths were removed. Femoral artery access sites were closed using percutaneous suture-mediated closure devices, followed by standard manual compression and 6–8 h of pressure dressing to prevent hematoma.

### Endpoints

The primary endpoint was the procedural success of TEVAR. Procedural success was defined as the precise deployment of the main stent-graft at the target position and complete closure of the proximal primary entry tear. Meanwhile, the incidence of intra- and postprocedural complications [endoleak, stent-related rupture, and retrograde Stanford type A aortic dissection (RTAD)], the secondary intervention and mortality rate were observed. In addition, the aortic remodeling (postprocedural FL thrombosis) was evaluated.

### Statistical analysis

Statistical analysis was performed using SPSS software Version 26.0 (SPSS, Inc., Armonk, New York). The normality of distribution was assessed with the Kolmogorov–Smirnov test. Normally distributed quantitative data were presented as mea*n* ± standard deviation and compared using Student's *t*-test. Non-normally distributed quantitative data were expressed as median and interquartile range (IQR) and compared with the Mann–Whitney U test. Categorical variables are summarized as *n* (%) and compared using chi-square and Fisher's exact tests. Two-sided *P* < 0.05 were considered statistically significant.

## Results

### Patients’ characteristics

The overall study population comprised 153 patients, with 72 patients (47.1%) in the simulation group and 81 patients (52.9%) in the non-simulation group. The average age was 67.1 ± 9.1 years, and 66.0% of being male. No significant differences were observed in demographic characteristics or comorbidities between the two groups (all *P* > 0.05). Detailed baseline and preprocedural imaging characteristics are presented in Table 1. Regarding aortic arch morphology, Type I was the most common (75.2%), followed by Type II (20.9%) and Type III (3.9%). Importantly, no statistically significant differences were found between the simulation and non-simulation groups in the TL-to-FL PG [15.0 (IQR: 11.0, 18.0) mmHg vs. 15.0 (IQR: 12.0, 18.0) mmHg, *P* = 0.326], TL area [12.3 (IQR: 8.4, 15.3) mm^2^ vs. 13.2 (IQR: 10.0, 17.6) mm^2^, *P* = 0.066], FL area [4.2 (IQR: 2.8, 5.3) mm^2^ vs. 3.2 (IQR: 2.1, 6.1) mm^2^, *P* = 0.362], or FL area proportion [66.0 (IQR: 59.0, 73.0)% vs. 69.0 (IQR: 64.0, 76.0)%, *P* = 0.190].

**Table 1:** Baseline characteristics and preprocedural imaging assessments of patients with Stanford B aortic dissection undergoing thoracic endovascular aortic repair.

Variables	Overall cohort (*n* = 153)	Non-simulation group (*n* = 81)	Simulation group (*n* = 72)	*P*-value
Demographic characteristics				
Age, years	67.06 ± 9.10	66.89 ± 9.31	67.25 ± 8.93	0.807
Male, % (n)	66.0 (101)	67.9 (55)	63.9 (46)	0.601
Body mass index, kg/m^2^	23.3 (20.4, 26.8)	23.6 (20.6, 26.9)	23.1 (20.3, 26.6)	0.592
Systolic blood pressure, mmHg	186.0 (169.0, 205.0)	184.0 (168.0, 200.0)	190.5 (170.0, 0.679211.0)	0.161
Diastolic blood pressure, mmHg	98.0 (90.0, 107.0)	97.0 (90.0, 105.0)	100.0 (91.8, 110.0)	0.209
Comorbidities, % (n)				
Family history of aortic disease	2.6 (4)	2.5 (2)	2.8 (2)	1.000
Inflammatory disease	2.0 (3)	2.5 (2)	1.4 (1)	1.000
Diabetes	33.3 (51)	34.6 (28)	31.9 (23)	0.731
Hypertension	79.1 (121)	81.5 (66)	76.4 (55)	0.439
Hyperlipidemia	23.5 (36)	25.9 (21)	20.8 (15)	0.459
Coronary artery disease	24.2 (37)	24.7 (20)	23.6 (17)	0.876
Atrial fibrillation	8.5 (13)	9.9 (8)	6.9 (5)	0.516
Previous cardiovascular surgery	11.8 (18)	9.9 (8)	13.9 (10)	0.442
NYHA Class ≥ 3	90.2 (138)	92.6 (75)	87.5 (63)	0.745
Transthoracic echocardiography assessment				
Aortic arch type, % (n)				
Type I	75.2 (115)	76.5 (62)	73.6 (53)	0.679
Type II	20.9 (32)	18.5 (15)	23.6 (17)	0.426
Type III	3.9 (6)	4.9 (4)	2.8 (2)	0.874
Left ventricular ejection fraction, %	58.0 (53.0, 62.0)	58.0 (54.0, 64.0)	58.0 (52.0, 60.0)	0.302
True and false lumen PG, mmHg	15.0 (12.0, 18.0)	15.0 (12.0, 18.0)	15.0 (11.0, 18.0)	0.326
Computed tomography angiography				
TL area, mm^2^	12.6 (9.3, 16.6)	13.2 (10.0, 17.6)	12.3 (8.4, 15.3)	0.066
FL area, mm^2^	4.0 (2.4, 5.8)	3.2 (2.1, 6.1)	4.2 (2.8, 5.3)	0.362
Retrograde systolic FL blood flow, %	19.6 (30)	22.2 (18)	16.7 (12)	0.388
FL partial thrombosis, %	39.2 (60)	37.0 (30)	41.7 (30)	0.558
FL area proportion, %	68.0 (61.0, 75.0)	69.0 (64.0, 76.0)	66.00 (59.0, 73.0)	0.190
Aortic aneurysm, % (n)	30.7 (47)	33.3 (27)	27.8 (20)	0.457
Preprocedural ischemic symptoms, % (n)	17.6 (27)	18.5 (15)	16.7 (12)	0.764

NYHA, New York heart association; TL, true lumen; FL, false lumen.

### Procedural and clinical outcomes

Procedural details and pre-discharge clinical outcomes are summarized in Table 2. The simulation group demonstrated a higher procedural success rate than the non-simulation group (100.0% vs. 95.1%, *P* = 0.042) and a greater stent graft coverage length (24.57 ± 3.87 cm vs. 21.04 ± 3.28 cm, *P* = 0.033), while requiring fewer stents (1.20 ± 0.49 vs. 1.67 ± 0.75, *P* < 0.001). Notably, the simulation group had lower rates of endoleak (0% vs. 9.9%, *P* = 0.007), stent-related rupture (0% vs. 4.9%, *P* = 0.002), and RTAD (0% vs. 3.7%, *P* = 0.287), along with shorter procedure duration (103.0 ± 23.9 min vs. 134.0 ± 23.3 min, *P* < 0.001) and reduced contrast agent usage (183.0 ± 78.1 mL vs. 277.0 ± 103.4 mL, *P* < 0.001).

**Table 2:** Procedural details and in-hospital clinical outcomes of patients with Stanford B aortic dissection undergoing thoracic endovascular aortic repair.

Variables	Overall cohort (*n* = 153)	Non-simulation group (*n* = 81)	Simulation group (*n* = 72)	*P*-value
Procedural details				
Procedural success, % (n)	97.4 (149)	95.1 (77)	100 (72)	0.042
Preprocedural mechanical ventilation, % (n)	25.5 (39)	25.9 (21)	25.0 (18)	0.896
Stent numbers	1.43 ± 0.63	1.67 ± 0.75	1.20 ± 0.49	<0.001
Stent covering length, cm	22.63 ± 3.75	21.04 ± 3.28	24.57 ± 3.87	0.033
Endoleak, % (n)	5.2 (8)	9.9 (8)	0 (0)	0.007
Stent-related rupture, % (n)	2.6 (4)	4.9 (4)	0 (0)	0.002
RTAD, % (n)	2.0 (3)	3.7 (3)	0 (0)	0.287
Paraplegia, % (n)	0 (0)	0 (0)	0 (0)	N/A
Procedural duration, min	119.4 ± 28.2	134.0 ± 23.3	103.0 ± 23.9	<0.001
Contrast medium, mGy	232.8 ± 103.4	277.0 ± 103.4	183.0 ± 78.1	<0.001
In-hospital outcomes				
All-cause mortality, % (n)	2.6 (4)	3.7 (3)	0 (0)	0.248
Myocardial infarction, % (n)	2.0 (3)	2.5 (2)	1.4 (1)	1.000
Disabling stroke, % (n)	2.0 (3)	2.5 (2)	1.4 (1)	1.000
Life-threatening bleeding, % (n)	3.9 (6)	6.2 (5)	1.4 (1)	0.269
Major vascular complications, % (n)	4.6 (7)	7.4 (6)	1.4 (1)	0.164
Cardiogenic shock, % (n)	1.3 (2)	2.5 (2)	0 (0)	0.498
Sternal wound infection, % (n)	0 (0)	0 (0)	0 (0)	1.000
Acute kidney injury Staging ≥ 3, % (n)	3.3 (5)	4.9 (4)	1.4 (1)	0.437
Stent FL thrombosis extent, % (n)				
Complete thrombosis	70.6 (108)	79.0 (64)	61.1 (44)	0.015
Partial thrombosis	20.9 (32)	21.0 (17)	20.8 (15)	0.981
None	8.5 (13)	0 (0)	18.1 (13)	<0.001
ICU stay, days	0.6 (0.3, 1.4)	0.8 (0.5, 2.1)	0.3 (0.2, 1.0)	<0.001
Hospital stay, days	8.0 (6.0, 13.0)	12.0 (9.0, 14.0)	6.0 (5.0, 7.0)	<0.001

AD, aortic dissection; RTAD, retrograde Stanford type A aortic dissection; N/A, not applicable; FL, false lumen; ICU, intensive care unit.

In terms of clinical outcomes, the simulation group showed a lower rate of complete FL thrombosis (61.1% vs. 79.0%, *P* = 0.015) and better recovery profiles, including shorter intensive unit care stay [0.3 (IQR: 0.2, 1.0) days vs. 0.8 (IQR: 0.5, 2.1) days, *P* < 0.001] and shorter hospital stay [6.0 (IQR: 5.0, 7.0) days vs. 12.0 (IQR: 9.0, 14.0) days, *P* < 0.001]. No other significant differences in clinical outcomes were observed between the two groups (all *P* > 0.05).

### CFD evaluation

**Figure 2:**
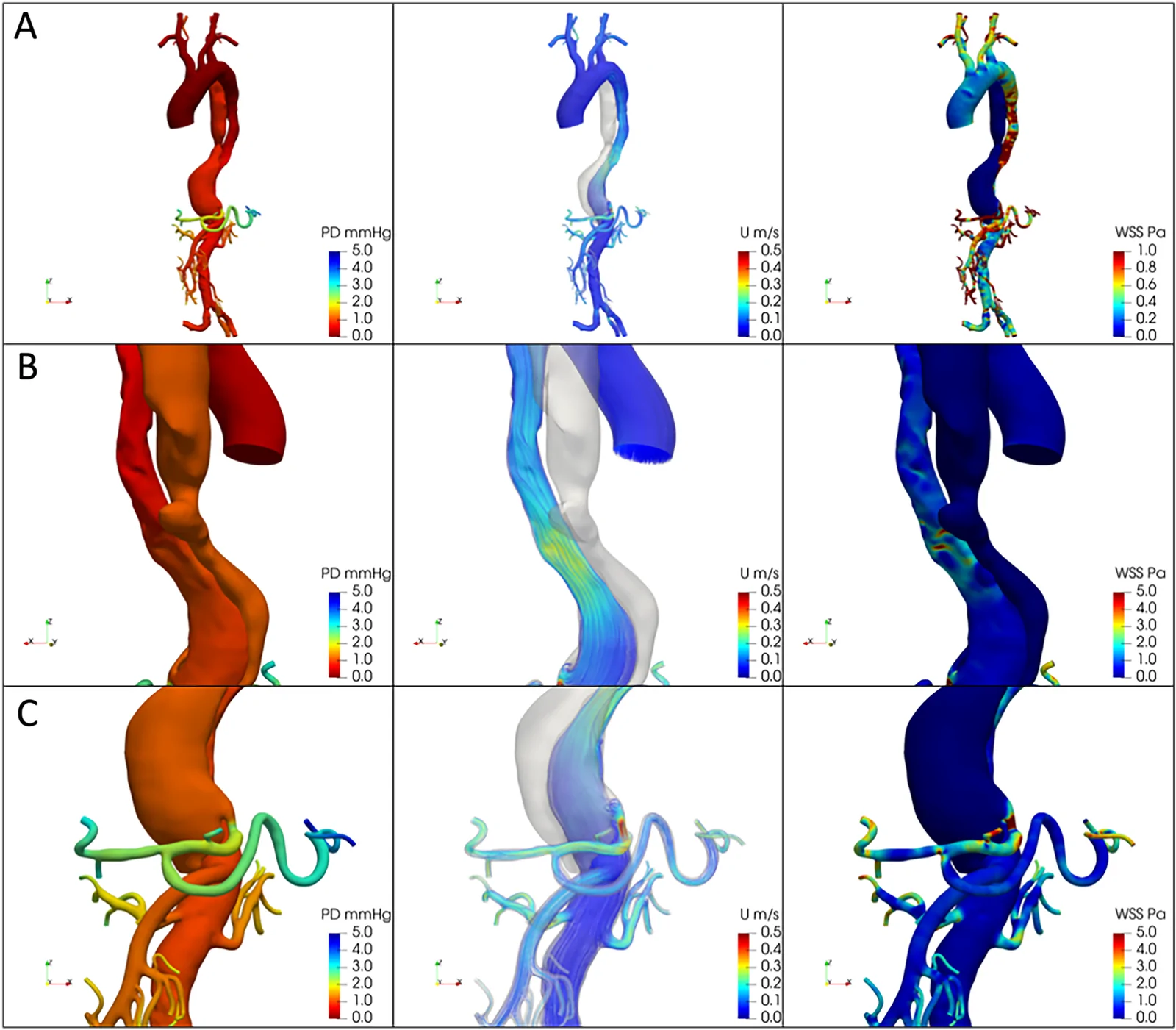
Preprocedural assessment based on computational hemodynamics. Each row shows, from left to right: the distribution of pressure gradient, flow velocity, and wall shear stress. **(A)** Overall view. **(B)** Local view of the descending aorta. **(C)** Local view of the celiac trunk.

**Figure 3:**
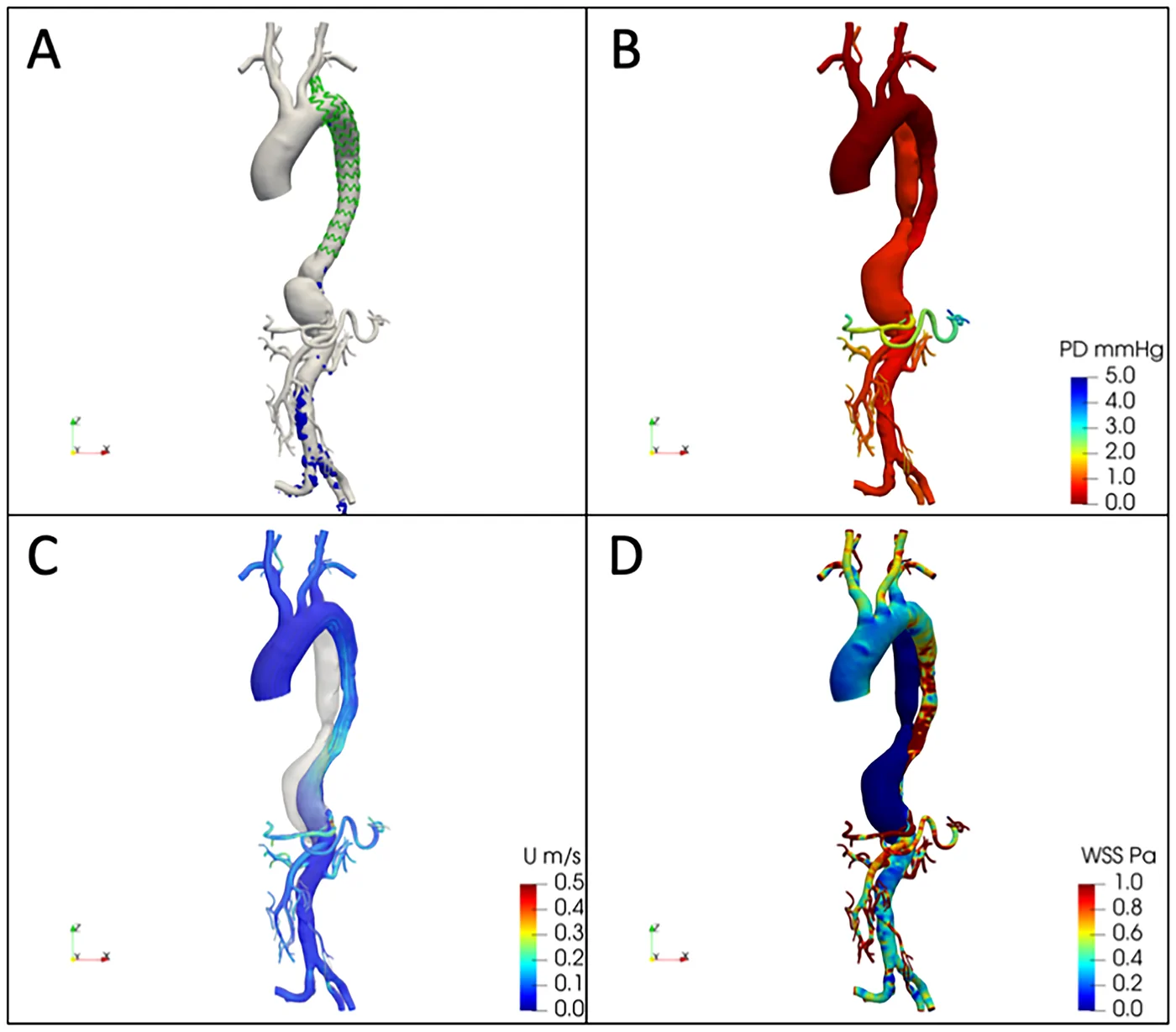
Computational simulation results. **(A)** Simulated stent deployment. **(B)** Simulated postprocedural pressure gradient distribution. **(C)** Simulated postprocedural flow velocity distribution. **(D)** Simulated postprocedural wall shear stress distribution.

**Figure 4:**
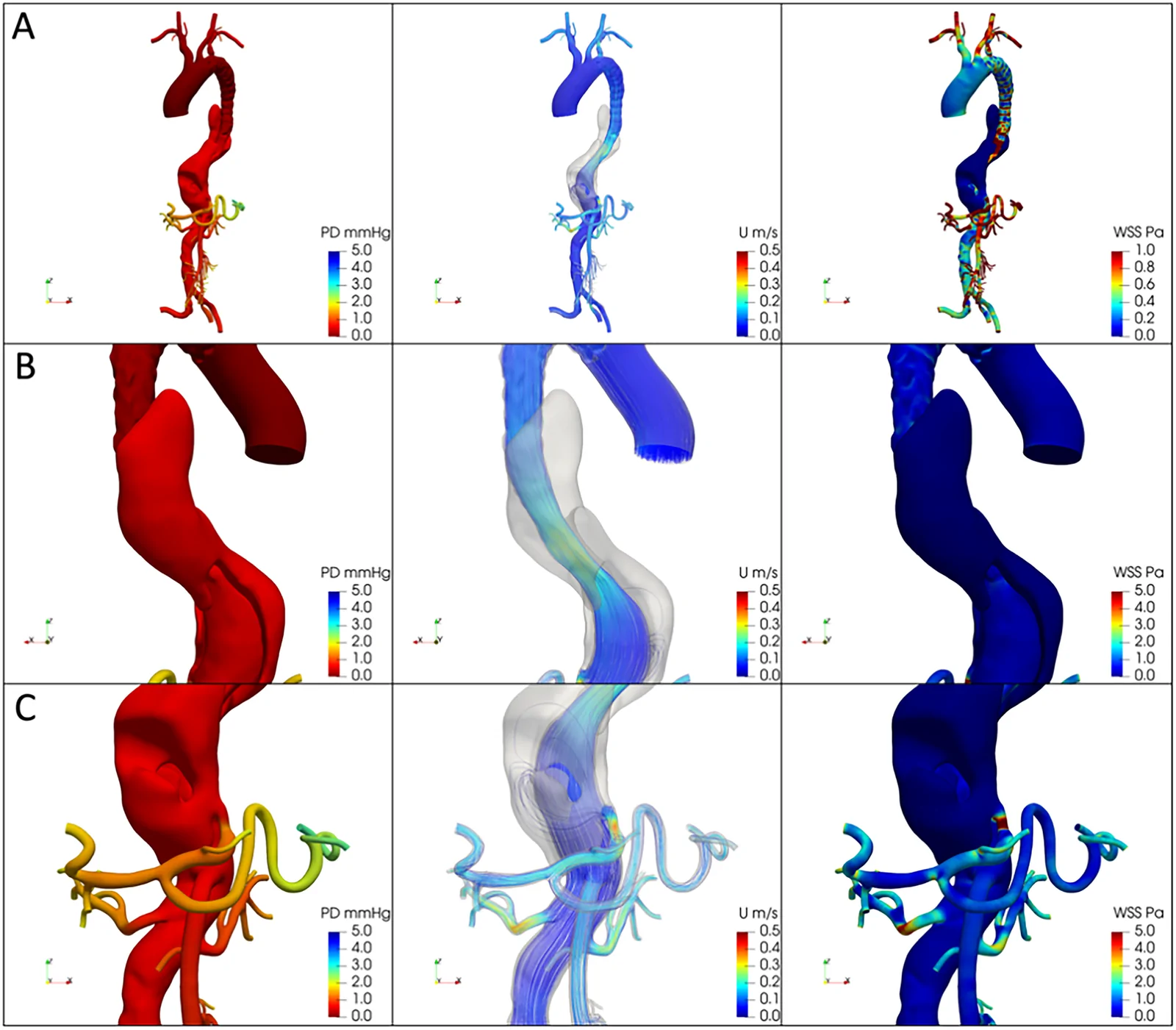
Postprocedural assessment based on computational hemodynamics. Each row shows, from left to right: the distribution of pressure gradient, flow velocity, and wall shear stress. **(A)** Overall view. **(B)** Local view of the descending aorta. **(C)** Local view of the celiac trunk.

Periprocedural CFD evaluation results are presented in Table 3. Pre-TEVAR CFD assessments were similar between the two groups, including the PG_max_, V_max_, and WSS_max_ in the FL (all *P* > 0.05) (Figure 2). Importantly, preprocedural stent implantation simulation was performed for all patients in the simulation group (Figure 3). Postprocedural hemodynamic performance in the simulation group was not only comparable to the simulated results (all *P* > 0.05) but also significantly better than that in the non-simulation group [PG_max_: 0.5 (IQR: 0.1, 1.5) mmHg vs. 2.4 (IQR: 1.3, 3.4) mmHg, *P* < 0.001; V_max_: 0.2 (IQR: 0.1, 0.4) m/s vs. 0.4 (IQR: 0.1, 0.8) m/s, *P* < 0.001; WSS_max_: 0.3 (IQR: 0.2, 1.0) Pa vs. 0.8 (IQR: 0.5, 2.1) Pa, *P* < 0.001] (Figure 4). Furthermore, the absolute error rates between simulated and postprocedural hemodynamic data in the simulation group were calculated as 5.2% for PG_max_, 3.1% for V_max_, and 6.3% for WSS_max_.

**Table 3:** Computational fluid dynamics assessments for patients with Stanford B aortic dissection undergoing thoracic endovascular aortic repair.

Variables	Overall cohort (*n* = 153)	Non-simulation group (*n* = 81)	Simulation group (*n* = 72)	*P*-value
Preprocedural assessment-FL				
PG_max_, mmHg	120.0 (95.0, 142.0)	122.0 (100.0, 154.0)	114.0 (94.0, 136.3)	0.089
V_max_, m/s	1.8 (1.3, 2.4)	2.0 (1.3, 2.4)	1.7 (1.2, 2.3)	0.086
WSS_max_, Pa	1.4 (0.8, 1.8)	1.5 (1.0, 2.1)	1.3 (0.7, 1.7)	0.209
Simulation assessment-FL				
PG_max_, mmHg	0.5 (0.1, 1.4)	N/A	0.5 (0.1, 1.4)	N/A
V_max_, m/s	0.2 (0.1, 0.3)	N/A	0.2 (0.1, 0.3)	N/A
WSS_max_, Pa	0.3 (0.1, 1.1)	N/A	0.3 (0.1, 1.1)	N/A
Postprocedural assessment-FL				
PG_max_, mmHg	1.5 (0.5, 2.7)	2.4 (1.3, 3.4)	0.5 (0.1, 1.5)	< 0.001
V_max_, m/s	0.3 (0.1, 0.5)	0.4 (0.1, 0.8)	0.2 (0.1, 0.4)	< 0.001
WSS_max_, Pa	0.6 (0.3, 1.4)	0.8 (0.5, 2.1)	0.3 (0.2, 1.0)	< 0.001

PG_max_, pressure gradient; V_max_, maximum velocity; WSS_max_, maximum wall shear stress.

## Discussion

In this study, we employed CFD technology to evaluate computer-simulated TEVAR for patients with TBAD and obtained the following key findings: First, by comparing simulation analyses with postprocedural hemodynamic data, we confirmed that computer simulation could accurately predict postprocedural blood flow status. Second, given the similar preprocedural hemodynamic profiles between the two groups, we demonstrated that computer-simulated assistance was associated with a higher procedural success rate and lower unadjusted incidence of complications. Furthermore, comparison of pre-discharge clinical outcomes revealed that the simulation group achieved more favorable prognosis, including performance in aortic remodeling.

Biomechanical factors play a critical role in the initiation and progression of TBAD. While TEVAR is increasingly applied in TBAD patients, interventional physicians often overlook the variability in vascular tissue mechanical properties and hemodynamic factors across individuals. On one hand, treatment planning frequently relies solely on aortic morphology for stent graft deployment, without accurate assessment of postoperative hemodynamic improvement. On the other hand, endograft implantation typically requires at least a 15 mm healthy proximal landing zone to ensure safety and stability (9). Unfortunately, over 40% of TBAD cases present with an insufficient proximal landing zone due to TL starting point being less than 15 mm from the left subclavian artery (10). Therefore, timely and accurate assessment of the true hemodynamic conditions within the vessel wall is particularly important.

In the field of CFD, preprocedural analysis of imaging data from specific patients with TBAD allows simulation of stent graft deployment and prediction of postprocedural outcomes. Such simulations help anticipate clinical outcomes and provide procedural guidance prior to intervention. In pre-TEVAR assessment, Yu et al. (11) developed a finite element model simulating AD formation and found that heterogeneity in aortic interlayer fibers is a key factor in rapid dissection propagation. Further, Bäumler et al. (12) innovatively proposed a comprehensive AD numerical model and demonstrated that its activity influences local hemodynamics. In periprocedural planning, some researchers have simulated TEVAR by covering the primary entry tear, showing that the procedure effectively reduces FL pressure, enhances wall stability, and preserves branch perfusion—suggesting potential reduction in adverse outcomes such as FL expansion and aortic rupture (13). Morris et al. (14) developed a model simulating physiological flow conditions in both TLs and FLs. This model showed less than 3% morphological discrepancy compared to CT data and, by monitoring flow patterns and pressure distributions across 12 cross-sections via echocardiography, could be used to evaluate the efficacy of open, endovascular, or hybrid surgical strategies. In postprocedural evaluation, Menichini et al. (15) demonstrated through computational modeling that areas of low WSS correlate with thrombus formation, suggesting that low WSS regions favor thrombosis. Similarly, Abazari et al. (16) performed numerical simulations using three-element Windkessel model boundary conditions at each outlet and indicated that reduced time-averaged WSS (TAWSS) may prevent vessel wall rupture at the intimal tear.

However, previous studies have primarily used computer simulations to analyze hemodynamic mechanisms in TBAD, while the potential of this technology to play a decisive role in preprocedural strategy formulation has not been fully evaluated. This study represented the first prospective clinical research assessing the utility of periprocedural CFD in guiding TEVAR for patients with TBAD. Our results showed that simulation-derived hemodynamic parameters were consistent with postprocedural assessments [PG_max_: 0.5 (IQR: 0.1, 1.4) mmHg vs. 0.5 (IQR: 0.1, 1.5) mmHg; V_max_: 0.2 (IQR: 0.1, 0.3) m/s vs. 0.2 (IQR: 0.1, 0.4) m/s; WSS_max_: 0.3 (IQR: 0.1, 1.1) Pa vs. 0.3 (IQR: 0.2, 1.0) Pa], indicating that computer simulation could accurately predict postprocedural hemodynamic status. The predicted near-complete exclusion of false lumen flow in Figure 3 occurs because covering the primary entry tear eliminates the dominant high-pressure systolic inflow vector; although distal re-entry tears remain open, the residual false lumen blood enters a stagnant, low-velocity state that favors subsequent thrombosis. Second, through high-quality preprocedural CFD simulation, the simulation group exhibited significantly better procedural performance than the non-simulation group. Notably, the simulation group achieved a procedural success rate of 100.0%, with no major complications such as endoleak, stent graft-induced rupture, or RTAD observed postprocedurally. Additionally, the simulation group exhibited a positive trend toward superior postprocedural aortic remodeling, reflected by a lower rate of complete FL thrombosis (61.1% vs. 79.0%). Crucially, given the unadjusted and non-randomized nature of this sequential cohort comparison, these improved clinical outcomes must be interpreted strictly as preliminary, exploratory associations rather than definitive causal evidence of enhanced prognosis, requiring validation in future adjusted trials. Finally, by analyzing the error rates between simulated and postprocedural hemodynamic data in the simulation group, we validated the reliability and stability of CFD technology.

The mechanistic explanation for the superior periprocedural and clinical outcomes observed in the simulation group directly stems from how the preprocedural computational framework altered clinical decision-making. Rather than relying strictly on conventional static geometry. clinicians utilized interactive virtual stent-graft deployment to optimize graft selection. By evaluating fluid-structure interactions under simulated peak systolic pressure waves, the framework guided the selection of longer, precisely oversized single endografts (24.57 ± 3.87 cm), which significantly minimized the necessity for multi-stent nesting configurations (1.20 ± 0.49). Furthermore, the granular mapping of maximum pressure gradients and wall shear stress at the distal landing zone allowed operators to proactively secure an optimal seal, preventing undersizing-related migration or distal endoleaks without requiring trailing extension strategies. For proximal branch vessel management, the simulation confirmed that a standalone Zone 3 landing configuration achieved complete primary tear coverage while maintaining favorable physiological perfusion through the left subclavian artery, thereby streamlining the operative workflow, reducing procedural duration, and eliminating the need for complex adjunctive fenestrations or carotid-subclavian bypasses.

Looking forward, integrating advanced hemodynamic simulation into future commercial planning platforms represents a necessary software evolution. Standard planning tools currently used by endovascular specialists focus predominantly on static geometric measurements. Incorporating real-time computational parameters such as automated wall shear stress and pressure gradient mappings into commercial platforms would allow clinicians to virtually evaluate custom endograft configurations under simulated physiological stress prior to intervention. This paradigm shift will significantly refine patient selection by identifying high-risk tear propagation profiles and optimize landing zone customization, transforming complex modeling into an accessible clinical standard.

Beyond direct procedural optimization, the increasing reliance on patient-specific simulation models carries important educational implications for future training pathways. while preprocedural simulation reduces planning uncertainty, over-reliance on these models may introduce cognitive automation bias among trainees. Excessive dependency on digital twins might alter autonomous clinical decision-making and reduce an operator's adaptive readiness when managing unexpected intraoperative findings or real-time anatomical variations that diverge from idealized computational predictions. Therefore, simulation frameworks should serve as cognitive adjuncts rather than absolute replacements for standard tactile skill acquisition and real-world complication training.

### Study limitations

The main limitations of this study include its relatively small sample size and observational design. First, although our sequential, time-dependent group assignment effectively eliminated immediate selection biases, this non-randomized approach inherently introduces learning-curve effects and temporal bias. Unmeasured confounders, including temporal shifts in operator experience, device choices, and procedural factors, could influence the outcomes. Future randomized controlled trials adjusting for these major clinical and anatomical confounders are warranted to definitively substantiate its independent clinical value. Second, all primary clinical and remodeling outcomes are limited to the in-hospital period. This timeframe does not capture long-term metrics including true lumen expansion, false lumen regression, distal aortic enlargement, or late reintervention rates. Outpatient surveillance is ongoing; whether acute hemodynamic benefits translate into durable clinical outcomes remains a key focus of our future long-term prospective studies. Third, although computational simulation plays a significant role in cardiovascular disease research, the construction of accurate 3D models relies on high-fidelity imaging data and detailed vascular wall structural information. Inevitable discrepancies during image acquisition and model reconstruction may affect the accuracy of computational results. Fourth, our model assumed rigid vessel walls and simplified stent-graft material properties, limiting its ability to predict complex endograft behavior in highly tortuous aortas or capture patient-specific tissue compliance and wall anisotropy. Future versions must incorporate fluid structure interaction and personalized vascular biomechanics to improve fidelity. Additionally, the simplified computational framework used standardized nitinol properties ignoring device-specific radial force profiles and deployment behaviors. Therefore, comparing simulations with real-world observations improves their accuracy and reliability. Yet computational efficiency remains a challenge: building personalized precision models is time- and resource-intensive. Integrating machine learning offers a promising path to accelerate simulations and enable personalization. Finally, the human circulatory system is inherently multidimensional and dynamically interacts with other organ systems; its complex pathophysiology makes accurate mechanistic modeling difficult. Ongoing refinement of computational models is essential to bridge the gap between theoretical insights and clinical needs.

## Conclusions

In this prospective observational study, patient-specific computational simulation of TEVAR for TBAD was associated with postprocedural hemodynamic profiles that aligned with simulated predictions, and showed favorable procedural trends and short-term clinical profiles. These exploratory findings suggest that CFD-based analysis may serve as a valuable adjunct for refining preprocedural planning in patients undergoing TEVAR. However, given the observational and unadjusted design, these results should be considered preliminary, and randomized controlled trials are warranted to establish definitive causal evidence.

## Data Availability

The original contributions presented in the study are included in the article/Supplementary Material, further inquiries can be directed to the corresponding author.
